# Case Report: Benralizumab combined with a steroid-sparing strategy in a case of severe eosinophilic granulomatosis with polyangiitis

**DOI:** 10.3389/fimmu.2026.1820718

**Published:** 2026-06-17

**Authors:** Nanxi Zhou, Chunxiao Fang, Min Feng, Chang Dong

**Affiliations:** Department of Respiratory and Critical Care Medicine, First Affiliated Hospital of Dalian Medical University, Dalian, China

**Keywords:** ANCA-negative, benralizumab, eosinophilic granulomatosis with polyangiitis, interstitial lung disease, steroid-sparing strategy

## Abstract

**Background:**

Eosinophilic granulomatosis with polyangiitis (EGPA) is a rare anti-neutrophil cytoplasmic antibody (ANCA)-associated vasculitis characterized by eosinophilic inflammation and necrotizing vasculitis. Benralizumab, an anti-IL-5Rα monoclonal antibody that directly targets eosinophils, was approved for EGPA in China in December 2025. We report an early real-world experience of benralizumab combined with corticosteroids in a patient with severe ANCA-negative EGPA.

**Case presentation:**

A 73-year-old male with a history of childhood asthma, chronic rhinosinusitis, CKD stage 4 presented with recurrent pulmonary infiltrates, progressive interstitial lung disease, severe eosinophilia (peak 12.90×10^9^/L), diffuse skin lesion and new-onset arrhythmia. After systematic differential diagnosis, EGPA was diagnosed according to the 2022 ACR/EULAR criteria (score 7). BALF and blood testing detected nucleic acids of *Pneumocystis jirovecii* and CMV. He was treated with methylprednisolone 40 mg/day and subcutaneous benralizumab 30 mg every 4 weeks. Concurrently, empirical preemptive anti-infective therapy (caspofungin for *Pneumocystis jirovecii*, ganciclovir for CMV) was initiated. At 4-week follow-up, prednisone was tapered to 30 mg/day; dyspnea and rash markedly improved, eosinophils decreased to 0, IgE fell from 1,860 to 519 IU/mL, chest CT showed significant resolution of infiltrates, and pulmonary function improved from severe to mild restrictive impairment. At 3 months, prednisone was further tapered to 25 mg/day in combination with benralizumab, and the patient remained stable without disease relapse or acute infection.

**Conclusion:**

This case represents an early real-world application of benralizumab in a high-risk ANCA-negative EGPA patient following its approval in China. The findings suggest that benralizumab, used as an adjunctive steroid-sparing agent, may facilitate rapid corticosteroid tapering in selected vulnerable patients with infection risk. Long-term follow-up and studies are needed to validate these findings.

## 1 Introduction

Eosinophilic granulomatosis with polyangiitis (EGPA) is a rare anti-neutrophil cytoplasmic antibody (ANCA)-associated vasculitis characterized by eosinophilia, eosinophilic infiltration, and necrotizing inflammation of small-to-medium-sized vessels. ANCA status serves as an important basis for identifying distinct clinical phenotypes. ANCA-positive patients accounting about 20%-40% of patients are more prone to vasculitic manifestations (e.g., glomerulonephritis, neuropathy), whereas ANCA-negative patients more commonly present with pulmonary and cardiac involvement driven by type 2-mediated eosinophilic inflammation, though overlap exists (1). In December 2025, benralizumab was approved for EGPA in China (2). By targeting the IL-5 receptor α subunit, it directly induces eosinophil apoptosis, achieving rapid depletion (3). The MANDARA trial demonstrated non-inferiority to mepolizumab for remission induction in relapsed/refractory EGPA, with superior corticosteroid-sparing effects (41 % vs. 26 % complete oral corticosteroid discontinuation) (4). Sustained remission has been confirmed in a two-year extension study (5), and European real-world data reported higher remission rates with benralizumab at 12 months (6). We report a proof-of-concept clinical observation of benralizumab combined with corticosteroids in a high-risk ANCA-negative EGPA patient, where conventional high-dose glucocorticoids and immunosuppressants were considered potentially harmful due to advanced age, comorbidities, and infection risk. This case highlights the steroid-sparing potential of benralizumab in a high-risk population and provides insights into the diagnostic challenges of ANCA-negative EGPA.

## 2 Case presentation

### 2.1 Clinical history and timeline

A 73-year-old male was admitted with worsening dyspnea following one month of recurrent cough and sputum. His medical history included childhood asthma (long-term remission), chronic rhinosinusitis, hypertension, abdominal aortic aneurysm stent placement (8 months prior), bilateral renal artery stenosis (right severe, left mild), and stage 4 chronic kidney disease (baseline creatinine 197 μmol/L). He had a 40-pack-year smoking history and multiple drug allergies (sulfonamides, tetracycline, streptomycin, ambroxol and cephalosporin).

**Figure 1:**
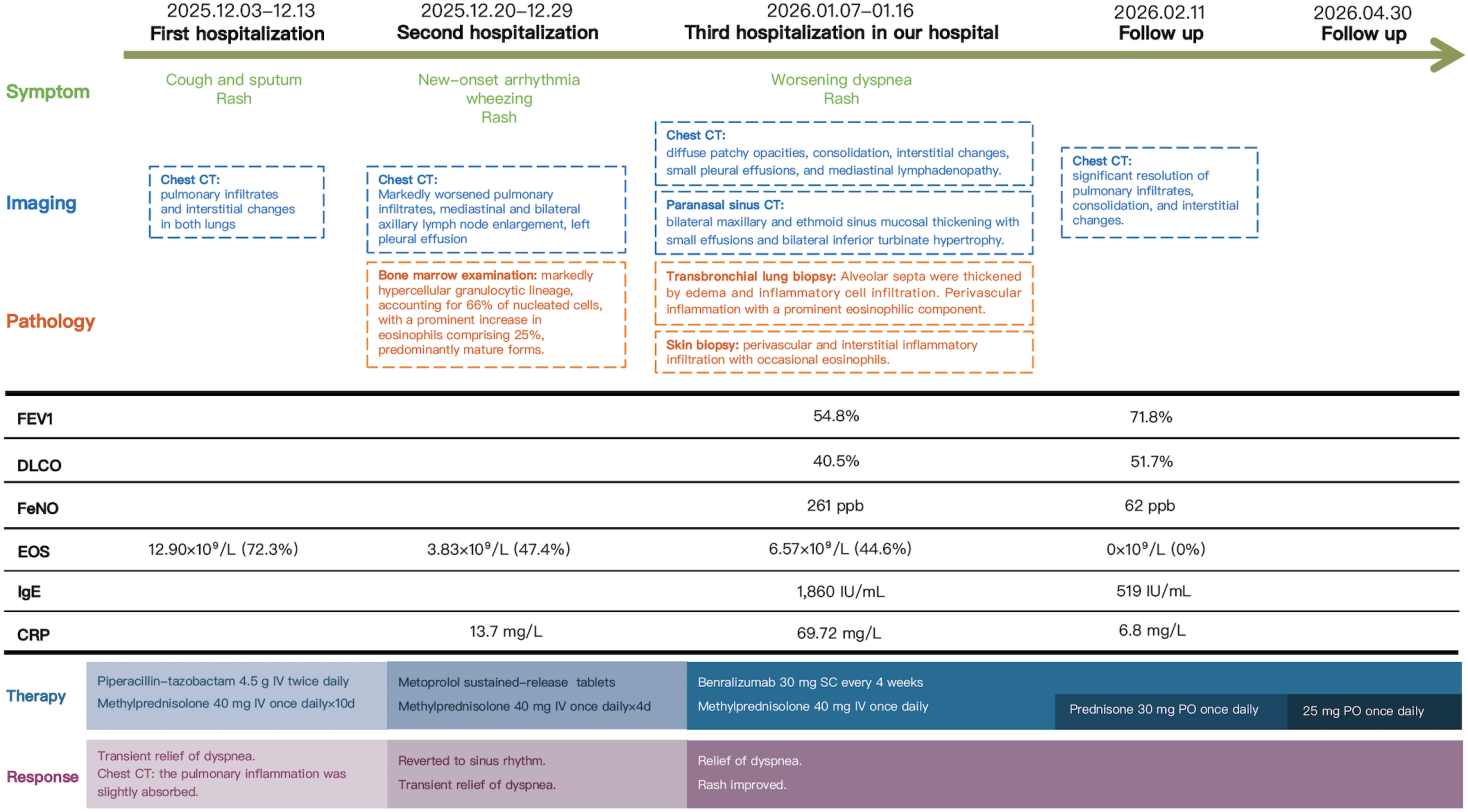
Timeline of the patient’s clinical course and workup. CT, computed tomography; FEV1, forced expiratory volume in 1 second; DLCO, diffusing capacity of the lungs for carbon monoxide; FeNO, fractional exhaled nitric oxide; ppb, parts per billion; EOS, eosinophils; IgE, immunoglobulin E; CRP, C-reactive protein; IV, intravenous; SC, subcutaneous; PO, orally.

**Figure 1** summarizes the timeline of the patient’s clinical course and workup, during which he was hospitalized three times within one month of symptom onset before receiving a definitive diagnosis. First hospitalization (2025-12-03): Presented with cough, sputum, and rash; marked eosinophilia (12.90×10^9^/L) and pulmonary infiltrates were noted, with partial improvement after antibiotics and corticosteroids. Second hospitalization (2025-12-20): Developed frequent premature ventricular contractions (2,844/24h, including pairs and triplets) and worsening dyspnea; After metoprolol sustained-release tablets and corticosteroids, arrhythmias resolved, but respiratory symptoms progressed. Third hospitalization (2026-01-07): Transferred to our hospital for management of respiratory failure; diagnosed with ANCA-negative EGPA.

### 2.2 Physical examination and initial assessment

**Figure 2:**
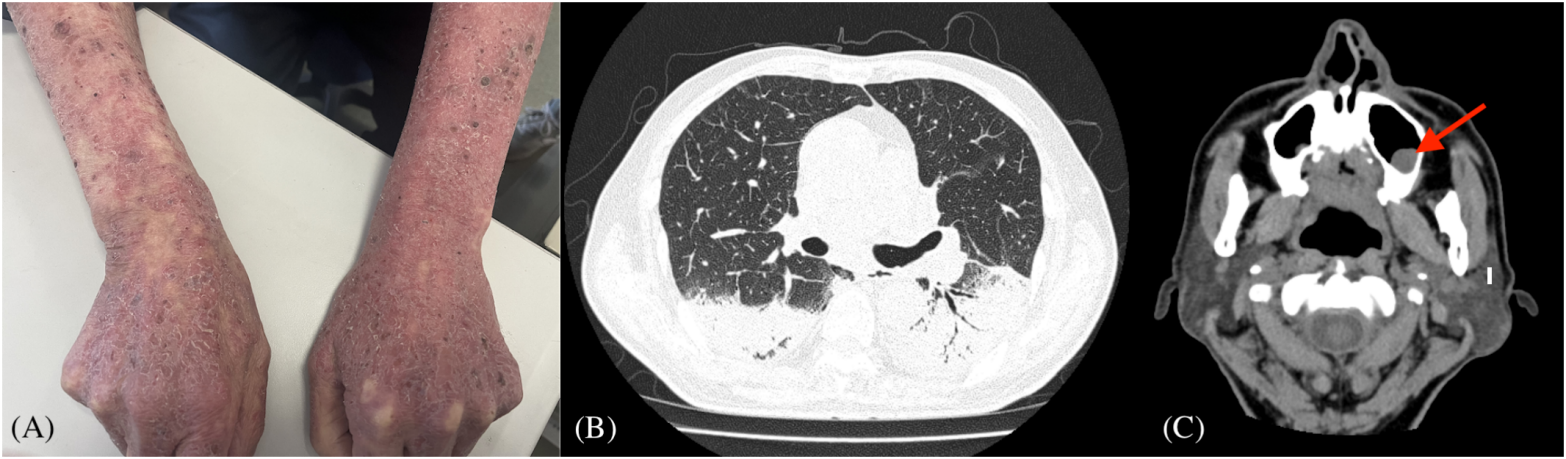
Multisystem involvement in a patient with ANCA-negative eosinophilic granulomatosis with polyangiitis (EGPA). **(A)** Extensive erythematous, scaly, crusted papules and desquamation over the entire body. **(B)** Chest high-resolution computed tomography (CT) showing bilateral patchy consolidations and interstitial changes. **(C)** Paranasal sinus CT demonstrating maxillary sinus mucosal thickening with small effusions(red arrow), indicating bilateral maxillary sinusitis.

On admission, temperature was 37.0 °C, pulse 81 beats per minute, respiratory rate 19 breaths per minute, blood pressure 153/75 mmHg, and oxygen saturation 84% on room air. Physical examination revealed diffuse erythema and papules with ulceration and desquamation over the entire body (**Figure 2A**), and Velcro crackles at both lung bases on auscultation. Cardiac and abdominal examinations were unremarkable. Key laboratory findings on admission were as follows. White blood cell count was 14.73×10^9^/L with eosinophilia (6.57×10^9^/L, 44.6%; reference range: 0.02-0.52×10^9^/L, 0.4%-8.0%). Neutrophils were 7.31×10^9^/L (49.6%), hemoglobin 122 g/L, and platelets 220×10^9^/L. C-reactive protein (CRP) was elevated at 69.72 mg/L (reference: 0–6 mg/L) and procalcitonin (PCT) at 1.29 μg/L (reference: 0-0.5 μg/L). Serum creatinine was 262 μmol/L (reference: 58-110 μmol/L), alanine aminotransferase (ALT) 126 U/L (reference: 7–40 U/L), aspartate aminotransferase (AST) 83 U/L (reference: 13–35 U/L), and albumin 25.4 g/L (reference: 35–55 g/L). Immunoglobulin E (IgE) was markedly elevated at 1,860 IU/mL (reference: 0–100 IU/mL) and lactate dehydrogenase (LDH) at 506 U/L (reference: 120–250 U/L). N-terminal pro-brain natriuretic peptide (NT-proBNP) was elevated at 2,750 ng/L (reference: 0–125 ng/L), indicating cardiac dysfunction, though troponin I was normal. Echocardiography showed left ventricular diastolic dysfunction (E/A=0.8) with preserved left ventricular ejection fraction (LVEF) of 62%. Electrocardiogram was unremarkable. Anti-Ku antibody was strongly positive (+++); anti-neutrophil cytoplasmic antibody (ANCA) panel (myeloperoxidase/proteinase 3), anti-cardiolipin antibody (ACA), anti-cyclic citrullinated peptide antibody (anti-CCP), creatine kinase (CK), creatine kinase-MB mass (CK-MB), and Krebs von den Lungen-6 (KL-6) were all negative. CD4+ T-cell count was mildly decreased at 307/μL (reference: 410-1,120/μL). Infection screening revealed (1,3)-β-D-glucan, galactomannan, and Aspergillus IgG were negative; sputum culture yielded no pathogens. Whole blood EBV-DNA 5.52×10^3^ copies/mL, plasma EBV-DNA 6.50×10^2^ copies/mL, and CMV-DNA 7.98×10^2^ copies/mL (reference: <5.00×10^2^ copies/mL for all). Targeted next-generation sequencing (tNGS) of bronchoalveolar lavage fluid (BALF) also detected *Pneumocystis jirovecii* (431 sequences), CMV (14,573 sequences) and EBV (886,351 sequences). Chest high-resolution computed tomography (HRCT) (**Figure 2B**) showed diffuse patchy opacities, consolidation, interstitial changes, small pleural effusions, and mediastinal lymphadenopathy. Paranasal sinus CT revealed bilateral maxillary and ethmoid sinusitis (**Figure 2C**). Pulmonary function tests demonstrated severe restrictive impairment [total lung capacity (TLC) 55.7% predicted, forced expiratory volume in 1 second (FEV1) 54.8% predicted] with reduced diffusion capacity (diffusing capacity of the lungs for carbon monoxide [DLCO] 40.5% predicted). Fractional exhaled nitric oxide (FeNO), a marker of eosinophilic airway inflammation (elevated ≥50 parts per billion [ppb]), was markedly elevated at 261 ppb.

### 2.3 Pathological and hematological findings

**Figure 3:**
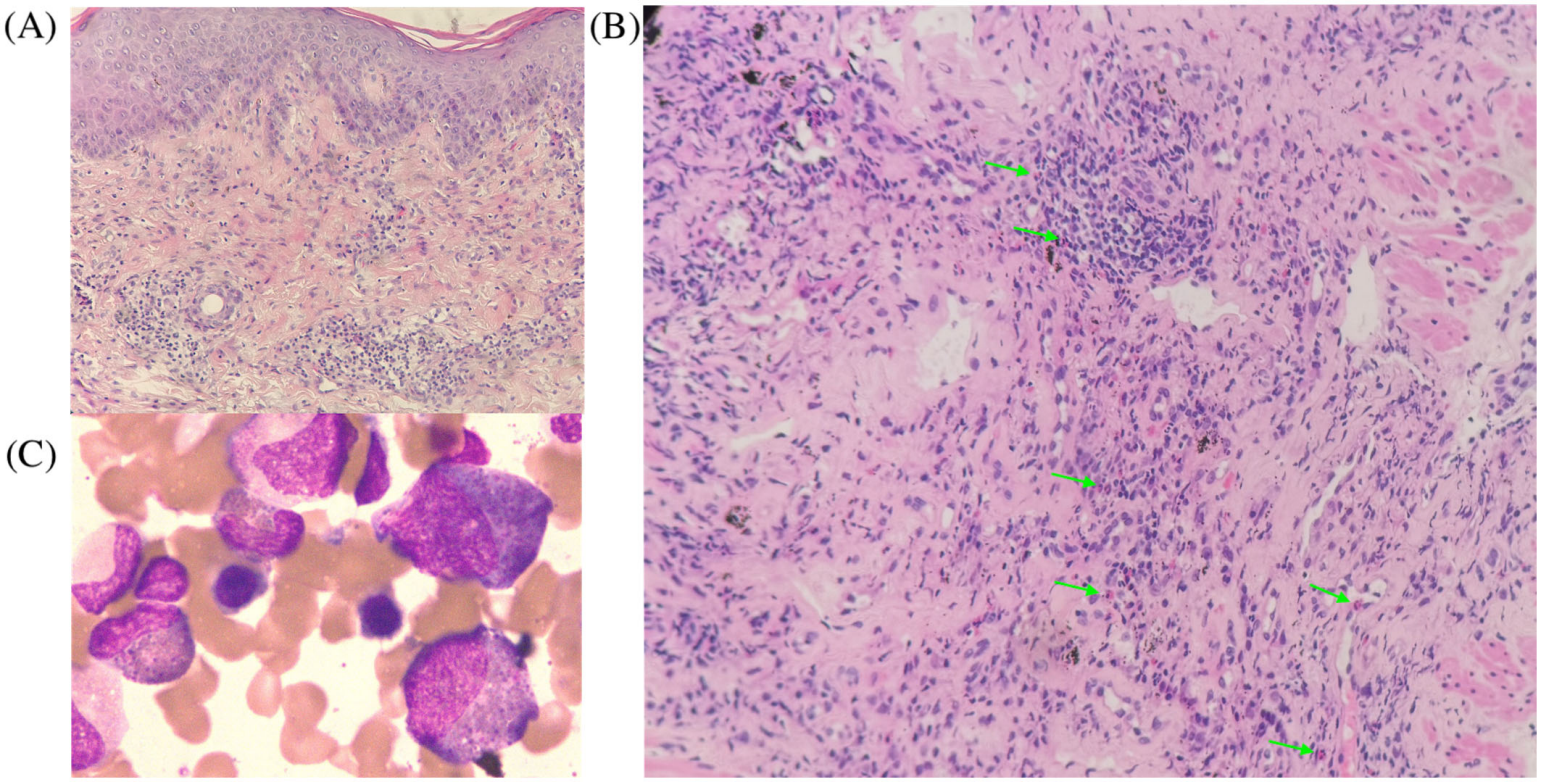
Pathological and bone marrow morphological findings. **(A)** Skin lesion biopsy (H&E stain): Perivascular and interstitial inflammatory infiltration with scattered eosinophils. **(B)** Transbronchial lung biopsy (H&E stain): Non-necrotizing perivascular inflammation with prominent mature eosinophilic infiltration (green arrows) and alveolar septal fibrosis. **(C)** Bone marrow smear (Wright-Giemsa stain): Marked eosinophilia, predominantly mature forms.

**Figure 4:**
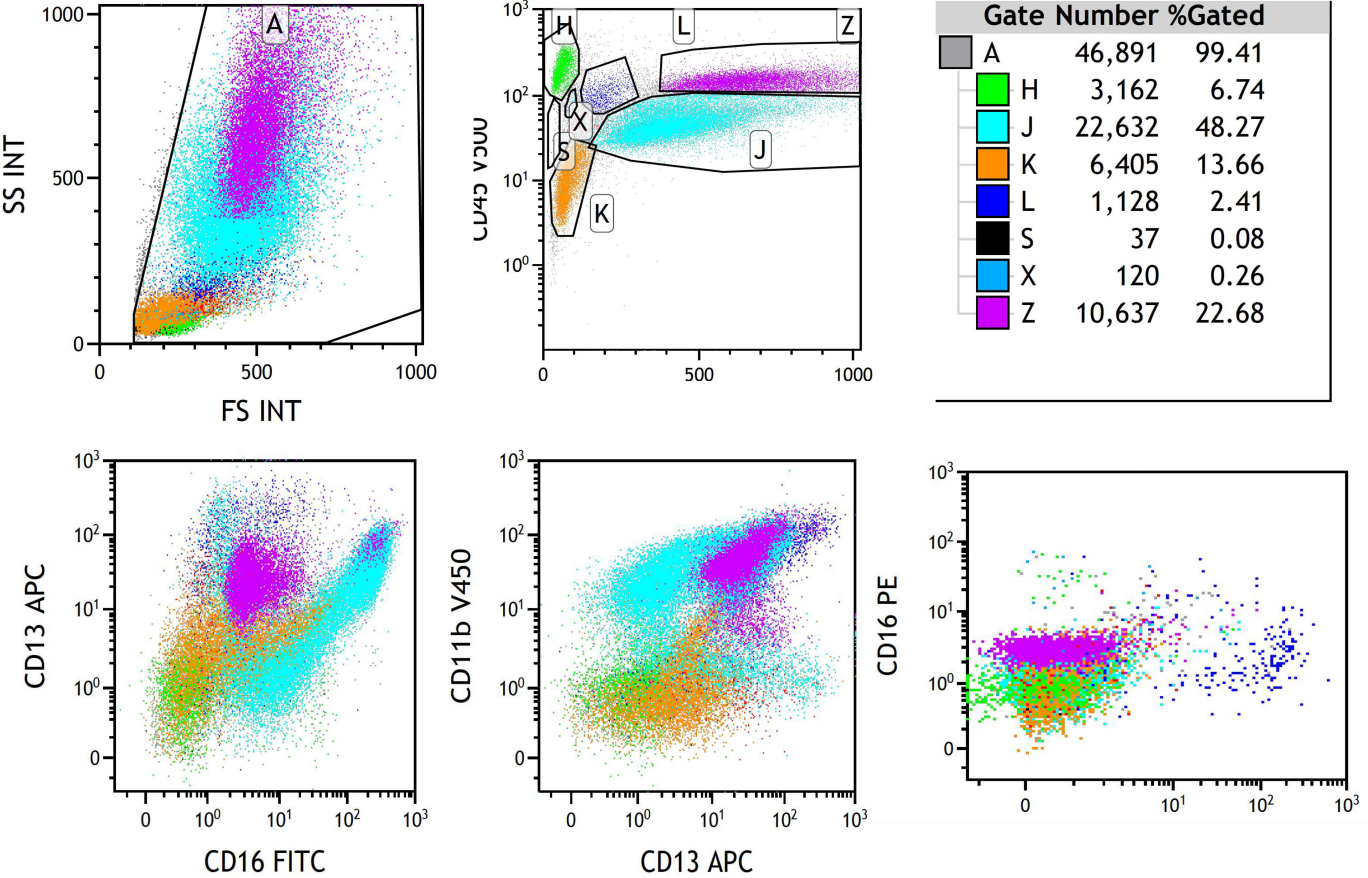
Bone marrow immunophenotyping findings. Flow cytometric analysis revealed marked eosinophilia, with eosinophils comprising 22.68% of nucleated cells. Granulocytes exhibited dysregulated antigen expression, characterized by elevated CD13, reduced/aberrant CD16, and imbalanced CD11b/CD13 co-expression. Normal blast count (CD34 + 0.47%); no aberrant cross-lineage markers (e.g., CD56, CD7); preserved maturation trajectory; normal CD10 expression (see **Supplementary File** for detailed analysis), supporting a reactive eosinophilia.

Skin lesion biopsy (**Figure 3A**) showed perivascular and interstitial inflammatory infiltration with occasional eosinophils. Transbronchial lung biopsy (**Figure 3B**) revealed alveolar septal fibrosis with eosinophilic and lymphocytic infiltration. Bone marrow examination was performed to evaluate the underlying cause of marked eosinophilia. Bone marrow aspirate smear revealed hypercellular granulocytic lineage, accounting for 66% of nucleated cells, with a prominent increase in eosinophils comprising 25%, predominantly mature forms (**Figure 3C**). Bone marrow immunophenotyping showed that granulocytes comprised 48.27% of nucleated cells, with eosinophils accounting for 22.68%. Dysregulated expression of myeloid differentiation antigens was observed, characterized by elevated CD13, reduced/aberrant CD16, and imbalanced CD11b/CD13 co-expression (**Figure 4**). A comprehensive genetic panel testing for 56 leukemia-associated genes, including the three fusion genes associated with eosinophilic disorders (PDGFRA, PDGFRB, FGFR1), was negative. Karyotyping showed a normal 46,XY chromosome complement with no clonal abnormalities.

### 2.4 Diagnosis and differential diagnosis

According to the 2022 American College of Rheumatology/European League Against Rheumatism (ACR/EULAR) EGPA classification criteria (7), the patient scored 7 points (eosinophils ≥1×10^9^/L: +5; biopsy showing extravascular eosinophil infiltration: +2), supporting the diagnosis of EGPA. The Birmingham Vasculitis Activity Score (BVAS) was 15, indicating acute disease involving pulmonary (6 points), cutaneous (6 points), nose (1 point), and general manifestations (2 points) (8). The Five-Factor Score (FFS) was 2 (age >65 years, serum creatinine >150 μmol/L), confirming severe EGPA and predicting high mortality risk (9). The differential diagnosis was systematically considered. Aberrant expression of CD13/CD16 and CD11b/CD13 on bone marrow granulocytes raised suspicion of clonal hypereosinophilic disorder (HES), but a normal karyotype, negative PDGFRA/PDGFRB/FGFR1 fusion genes, and absence of other clonal markers excluded HES. Systemic inflammation in ANCA-associated vasculitis has been reported to induce reactive myeloid phenotypic changes, including altered CD16 and CD11b expression (10, 11); this immunophenotype might be a reactive phenomenon associated with systemic inflammation in EGPA. This observation underscores the well-recognized difficulty in distinguishing EGPA from HES, particularly between ANCA-negative EGPA and idiopathic hypereosinophilic syndrome (I-HES). Of note, overlap cases between these two disorders may exist (12). Drug reaction with eosinophilia and systemic symptoms (DRESS) was considered because of the rash and eosinophilia, but DRESS typically develops 2-8 weeks after culprit drug exposure and resolves after drug withdrawal, whereas our patient’s rash preceded any suspected medication. Primary eosinophilic pneumonia could not account for the extrapulmonary involvement (sinusitis, skin lesions). Parasitic infection was excluded by negative stool examinations and lack of epidemiological risk factors. Other ANCA-associated vasculitides are unlikely given negative ANCA and inability to account for profound eosinophilia. Taking all clinical, laboratory, histopathological, and genetic findings together, the patient was most consistent with a diagnosis of ANCA-negative EGPA.

### 2.5 Treatment and follow-up

The patient had laboratory evidence suggesting increased susceptibility to opportunistic infections.

The *Pneumocystis jirovecii* sequence count with negative (1,3)-β-D-glucan was more consistent with colonization; the CMV DNA load (7.98×10^2^ copies/mL) indicated low-level reactivation without tissue-invasive disease; and borderline-positive EBV DNA reflects low-level reactivation in a past infection setting without clinical significance. Given his advanced age, CKD stage 4, conventional high-dose glucocorticoids and immunosuppressants (e.g., cyclophosphamide) were considered potentially harmful. Therefore, EGPA treatment was initiated with methylprednisolone 40 mg/day (approximately 0.5 mg/kg) combined with subcutaneous benralizumab 30 mg every 4 weeks. Given that the patient was expected to receive long-term moderate-to-high-dose glucocorticoid therapy for severe EGPA, his risk of developing *Pneumocystis jirovecii* pneumonia (PJP) was increased. PJP carries a high mortality rate, with a 30-day mortality of up to 52.7% and a 90-day mortality of 67.9% in immunocompromised non-HIV patients (13). Moreover, the patient was sulfa-allergic, which precluded first-line trimethoprim/sulfamethoxazole prophylaxis. Therefore, we initiated caspofungin (50 mg daily) as second-line PJP prophylaxis and ganciclovir (100 mg daily) as pre-emptive therapy for CMV. We acknowledge that echinocandins are not guideline-recommended first-line prophylactic agents for PJP (14); this management decision reflected individual clinical considerations in a patient with sulfonamide allergy and multiple comorbidities. The patient showed rapid clinical improvement with better oxygenation after treatment. Before discharge, CRP decreased to 18.2 mg/L, eosinophils to 0.32×10^9^/L (2.1%), and IgE to 890 IU/mL. At 4-week follow-up, prednisone was tapered to 30 mg/day. He was afebrile with improved exercise tolerance, and oxygen saturation remained 95-98% on room air. Skin rash had mostly resolved. Eosinophils became undetectable; IgE fell to 519 IU/mL, and CRP decreased to 6.8 mg/L. Chest HRCT showed significant resolution of infiltrates, consolidation, and interstitial changes. Pulmonary function tests improved to mild restrictive impairment (TLC 72.8% predicted, FEV1 71.8% predicted, DLCO 51.7% predicted), and FeNO decreased to 62 ppb reflecting reduced airway inflammation. At 3-month follow-up, the patient remained stable without EGPA relapse or opportunistic infection, and prednisone was further tapered to 25 mg/day in combination with continued benralizumab 30 mg every 4 weeks.

## 3 Discussion

We report a 73-year-old patient with severe ANCA-negative EGPA, profound eosinophilia, progressive ILD, cutaneous involvement, stage 4 CKD, in whom high-dose glucocorticoids and conventional immunosuppressants were considered potentially harmful. The patient was treated with benralizumab combined with a steroid-sparing strategy and achieved clinical improvement. We acknowledge that glucocorticoids were the primary driver of the acute-phase improvement. The additional potential benefits of benralizumab may include: First, benralizumab was associated with rapid, targeted eosinophil depletion, which may help control Th2-driven eosinophilic inflammation (15). Second, the addition of benralizumab facilitates early tapering of corticosteroids, which may help reduce excessive immunosuppression and better maintain host immune competence.

Pulmonary involvement in EGPA extends beyond obstructive airway disease. While the 2022 ACR/EULAR criteria emphasize obstructive airway disease, recent studies indicate that ground-glass opacities and consolidation are the most frequent imaging manifestations, pathologically corresponding to eosinophilic pneumonia (EP) or organizing pneumonia (16, 17). Our patient’s findings were more consistent with an EP-predominant pattern: chest HRCT showed bilateral patchy opacities and consolidation with interstitial changes. Transbronchial lung biopsy revealed prominent perivascular and interstitial eosinophilic infiltration with alveolar septal fibrosis, supporting EGPA-associated interstitial lung disease (ILD). Notably, the patient tested strongly positive for anti-Ku antibodies, which are associated with ILD. Recent studies report that the incidence of ILD is approximately 23% in anti-Ku-positive patients, with nonspecific interstitial pneumonia (NSIP) being the most common radiological pattern (31.8%-53%) (18, 19). Although our patient has no clinical features of connective tissue disease (CTD), and anti-Ku positivity alone cannot fully explain his asthma, eosinophilia, or severe skin lesions, a possible overlap between EGPA and anti-Ku antibody-positive ILD cannot be excluded and warrants long-term follow-up.

Cutaneous involvement occurs in approximately 40%–60% of EGPA patients, mediated by direct eosinophil infiltration and small-vessel vasculitis (20). Our patient presented with very severe diffuse erythema, desquamation, pruritus, skin breakdown, and crusting. Skin biopsy revealed perivascular and interstitial inflammatory infiltration with occasional eosinophils—a pattern consistent with the ANCA-negative EGPA phenotype, which is characterized predominantly by tissue injury rather than frank vasculitis (21).

Cardiac involvement in EGPA is common, with higher rates reported in ANCA-negative patients (22). Arrhythmia is a common early manifestation—including premature ventricular contractions, atrial arrhythmias, and atrioventricular block (23, 24). Our patient developed new-onset ventricular arrhythmia coinciding with peak disease activity, along with elevated NT-proBNP and diastolic dysfunction on echocardiography. The arrhythmia resolved with anti-inflammatory therapy without antiarrhythmic drugs, suggesting a possible subclinical myocardial involvement. However, cardiac magnetic resonance or endomyocardial biopsy was not performed and definite cardiac involvement cannot be confirmed.

This study has several limitations. First, as a single real-world case observation, the role of benralizumab in clinical improvement remains to be further validated. Second, definite cardiac involvement could not be confirmed. Third, a possible overlap with anti-Ku-associated ILD remains uncertain. Fourth, the 3-month follow-up period is relatively short, which is insufficient to assess sustained remission, long-term steroid-sparing durability, ILD evolution, or disease progression. Longer observation and larger studies are needed to validate these preliminary findings.

## 4 Conclusion

In conclusion, this case suggests that benralizumab, as an adjunctive steroid-sparing agent, may facilitate eosinophil depletion and early corticosteroid tapering in high-risk EGPA patients. EGPA can be difficult to distinguish from HES and may occasionally overlap with other connective tissue diseases; therefore, clinicians should maintain a high index of suspicion and provide long-term follow-up for disease progression.

## Data Availability

The original contributions presented in the study are included in the article/**Supplementary Material**. Further inquiries can be directed to the corresponding author.
