# Supplementary material for: Case Report: Benralizumab combined with a steroid-sparing strategy in a case of severe eosinophilic granulomatosis with polyangiitis
Source: Front Immunol. 2026 Jun 17;17:1820718. doi: 10.3389/fimmu.2026.1820718 (PMC13318563; doi:10.3389/fimmu.2026.1820718)

mds liangyi-1 00068204 023

[AR]

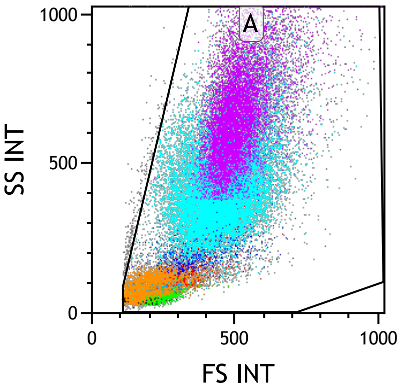

[A]

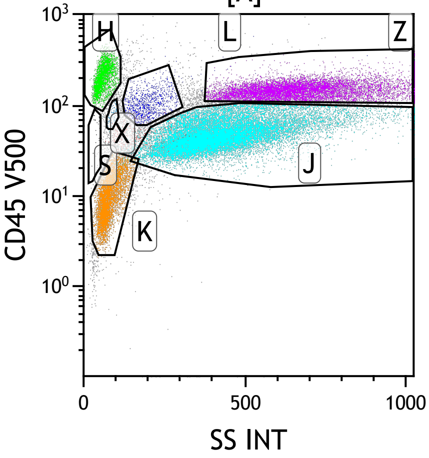

mds liangyi-1 00068204 023

Gate	Number	%Gated
A	46,891	99.41
H	3,162	6.74
J	22,632	48.27
K	6,405	13.66
L	1,128	2.41
S	37	0.08
X	120	0.26
Z	10,637	22.68

[B]

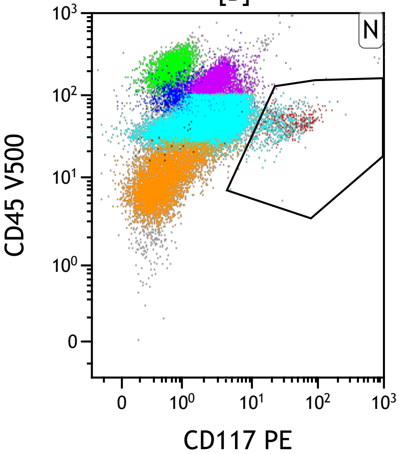

[N]

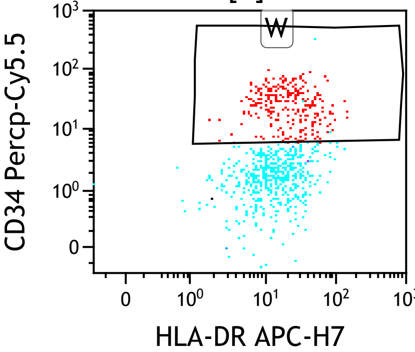

Gate	Number	%Total	%Gated
All	760	1.41	100.00
W	259	0.48	34.08

[W]

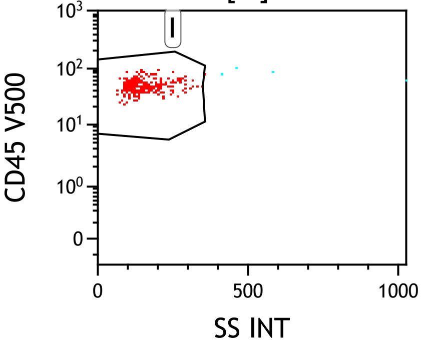

Gate	Number	%Total	%Gated
All	259	0.48	100.00
I	255	0.47	98.46

[A]

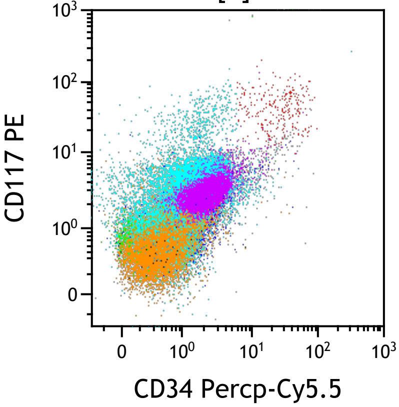

[A]

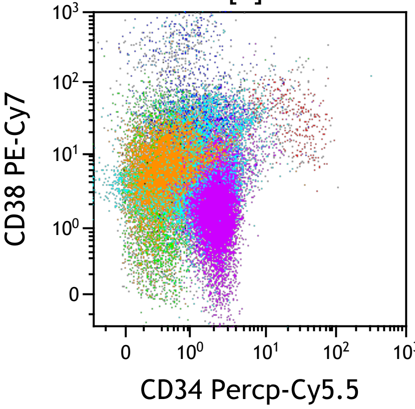

[B]

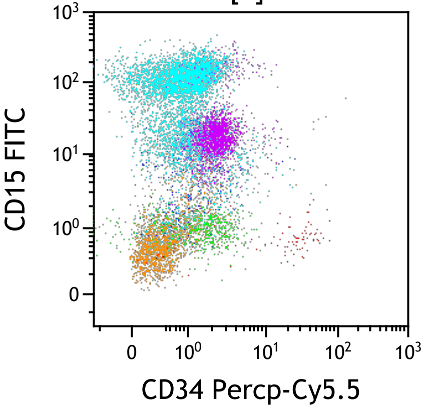

[A]

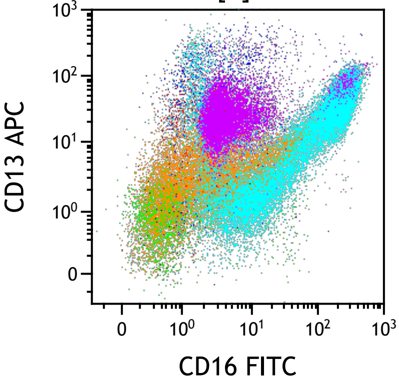

[A]

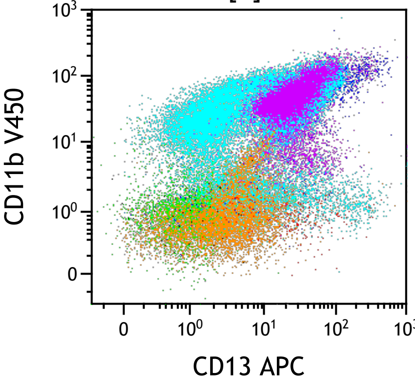

[A]

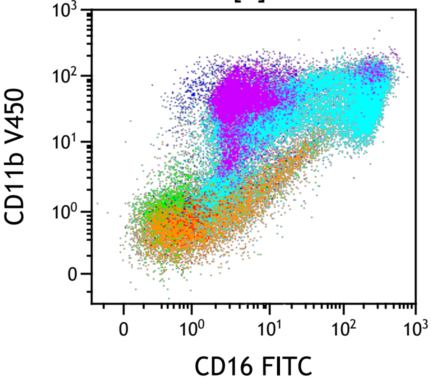

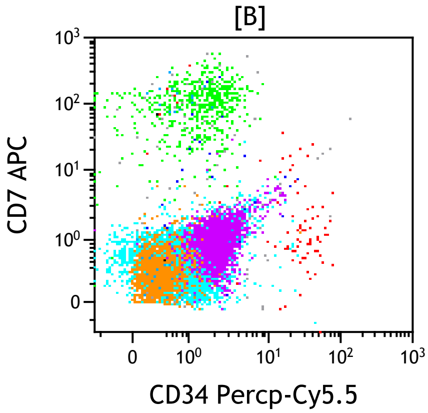

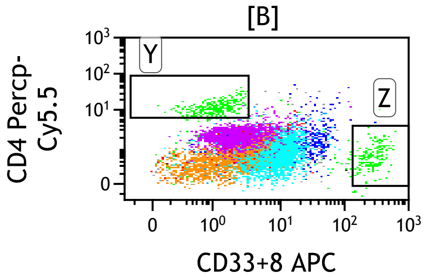

Gate	Number	%Total	%Gated
All	9,173	85.65	100.00
Y	316	2.95	3.44
Z	172	1.61	1.88

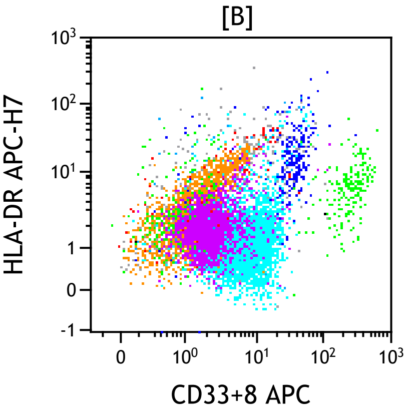

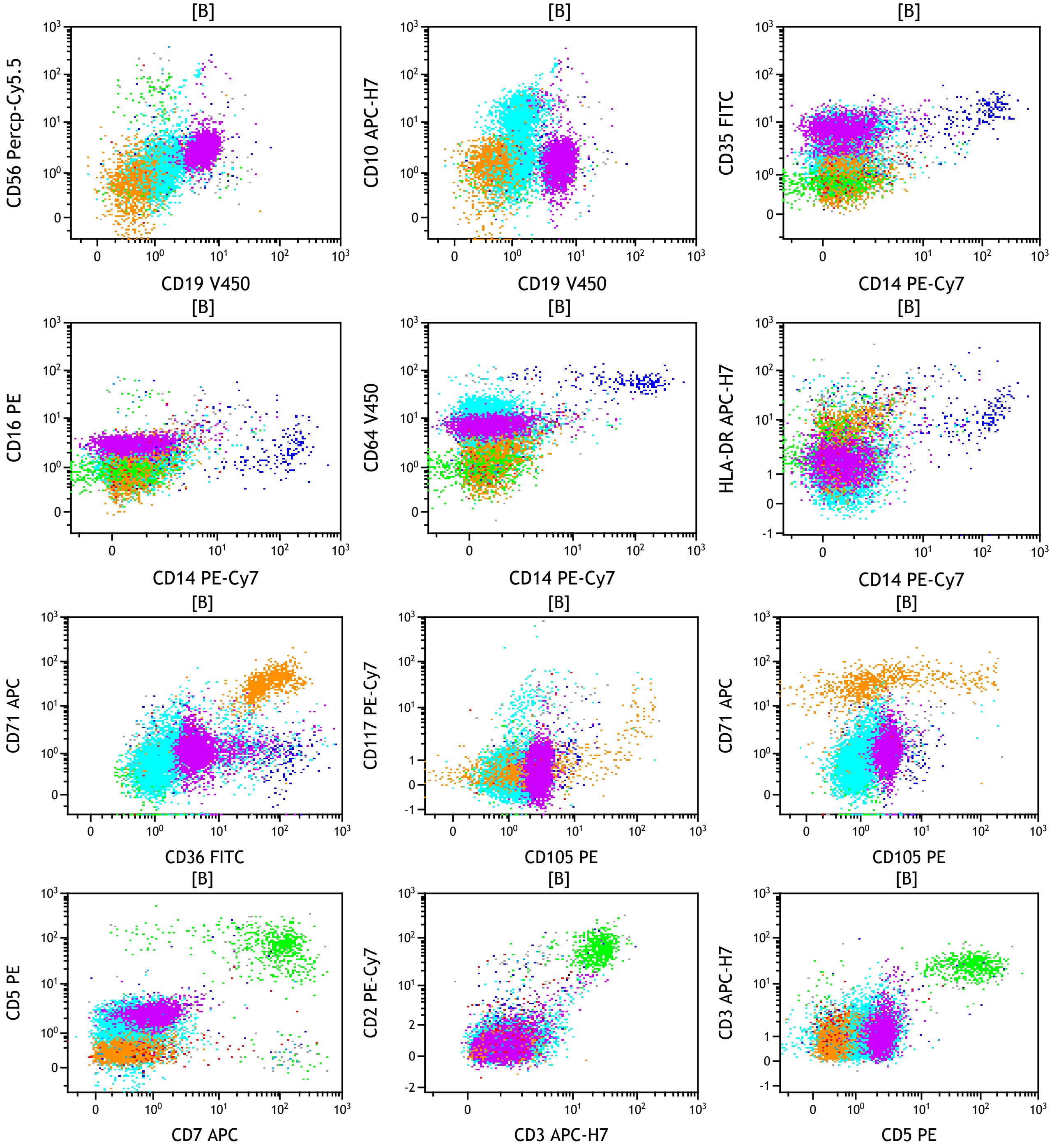

Supplement: Supplementary file 1 [file DataSheet1.pdf]
